# Multiple sclerosis and myelin basic protein: insights into protein disorder and disease

**DOI:** 10.1007/s00726-021-03111-7

**Published:** 2021-12-10

**Authors:** Vebjørn Martinsen, Petri Kursula

**Affiliations:** 1 Department of Biomedicine, University of Bergen, Jonas Lies vei 91, 5020 Bergen, Norway; 2 Biocenter Oulu and Faculty of Biochemistry and Molecular Medicine, University of Oulu, Aapistie 7, 90220 Oulu, Finland

**Keywords:** Myelin, Multiple sclerosis, Myelin basic protein, Disorder, Disease

## Abstract

Myelin basic protein (MBP) is an abundant protein in central nervous system (CNS) myelin. MBP has long been studied as a factor in the pathogenesis of the autoimmune neurodegenerative disease multiple sclerosis (MS). MS is characterized by CNS inflammation, demyelination, and axonal loss. One of the main theories on the pathogenesis of MS suggests that exposure to foreign antigens causes the activation of cross-reactive T cells in genetically susceptible individuals, with MBP being a possible autoantigen. While a direct role for MBP as a primary antigen in human MS is unclear, it is clear that MBP and its functions in myelin formation and long-term maintenance are linked to MS. This review looks at some key molecular characteristics of MBP and its relevance to MS, as well as the mechanisms of possible molecular mimicry between MBP and some viral antigens. We also discuss the use of serum anti-myelin antibodies as biomarkers for disease. MBP is a prime example of an apparently simple, but in fact biochemically and structurally complex molecule, which is closely linked to both normal nervous system development and neurodegenerative disease.

## Introduction

The word myelin stems from the Greek word for marrow (*myelos*), devised by the German pathologist Rudolf Ludwig Carl Virchow (1821–1902) in 1854 (Virchow 1854; Boullerne 2016), while the myelin structure had already been observed in the early eighteenth century (Van Leeuwenhoek 1719). The myelin sheath is a multilayered proteolipid membrane, which is involved in neural insulation and saltatory conduction of nerve impulses. It is, therefore, important in the function of both the central (CNS) and peripheral nervous system (PNS). Destruction of the myelin sheath causes neurodegeneration and conduction failure, as observed in demyelinating diseases, such as multiple sclerosis (MS) and acute disseminated encephalomyelitis (ADEM) in the CNS, and Guillain–Barré syndrome (GBS) and Charcot–Marie–Tooth disease (CMT) in the PNS. CMT is an inherited peripheral neuropathy, linked to mutations in dozens of different genes (Stavrou et al. 2021). The most common of these myelin disorders, and the focus of this short review, is MS.

MS is a multifactorial, autoimmune, demyelinating, neurodegenerative disease with an unknown pathogenesis (McGinley et al. 2021). It is characterized by CNS inflammation, demyelination, and axonal loss, as well as attempts at re-myelination by oligodendrocytes. One of the leading theories is that MS is caused by an aberrant immune response in genetically susceptible individuals (Fujinami and Oldstone 1985). MS is, thus, generally considered an autoimmune disease. Some research on the pathogenesis of MS has focused on the possible link between MS and an abundant protein found in CNS myelin, the myelin basic protein (MBP).

MBP is the second-most abundant protein in myelin, constituting 30% of the total CNS myelin protein. It was first isolated in the early 1960s (Einstein et al. 1962), being the most widely studied myelin protein in relation to MS. MBP, earlier called basic A1 protein, was first sequenced from bovine spinal cord and human myelin 50 years ago (Eylar 1970; Carnegie 1971; Eylar et al. 1971). It is an intrinsically disordered protein (IDP) (Harauz et al. 2009; Majava et al. 2010), lacking a well-defined globular structure, and it can change its conformation depending on its environment and interactions. Most importantly, upon binding to lipid bilayer surfaces, MBP folds into ⍺-helical structures and attaches tightly to the membrane (Harauz et al. 2009; Muruganandam et al. 2013; Raasakka et al. 2017). Upon interacting with its partner proteins, MBP likely forms short structured segments. The flexibility and adaptability of IDPs have been of growing interest in the field of structural biology, to a large extent due to an overall increasing focus on protein disorder and biological liquid–liquid phase separation. Being an IDP suggests that MBP could be a multifunctional protein (Tompa et al. 2005). It has been shown that MBP binds to and interacts with several other proteins, such as calmodulin and cytoskeletal proteins (Baryłko and Dobrowolski 1984; Chan et al. 1990; Majava et al. 2008; Harauz and Libich 2009; Smith et al. 2012), and it has been suggested to play a role in signaling pathways (Boggs 2006; Boggs et al. 2011; Vassall et al. 2015).

Extensive research has been done on both MBP and the possible role it plays as a source for autoantigenic epitopes in MS. It is largely undisputed that there are changes in the isoform composition and structure of MBP, as well as in compact myelin, during the pathogenesis of MS (Wood et al. 1996; Beniac et al. 1999; Boggs et al. 1999). The mechanisms of such changes are not clear at the molecular level. It is possible that an abnormal isoform composition of MBP leads to weakened membrane interactions and loosening of the rigid myelin structure. This may further lead to the observed anti-MBP immunoactivity as well as the presence of MBP in the cerebrospinal fluid (CSF). Whether the latter can be used as a biomarker of disease, is still under debate.

This short review will discuss some of the important aspects of MBP with regard to MS. It will take a brief look at the use of animal models for demyelinating disease and related challenges. Lastly, it will look at one of the leading theories today; the possible relationship between certain foreign antigens and the development of MS. The usefulness of MBP as a biomarker for MS is additionally discussed.

## Diagnostic criteria for multiple sclerosis

Clinically definite multiple sclerosis was defined by Poser et al*.* in “Diagnosis of Multiple Sclerosis: guidelines for research protocols” in 1983 as: two attacks with clinical evidence of separate lesions, or two attacks: with clinical evidence of one lesion and para-clinical evidence of another. The two attacks must be separated in space (different parts of CNS) and time (at least one month). The attack must last for at least 24 h (Poser et al. 1983).

Diagnosis of multiple sclerosis as defined by McDonald criteria was established in 2001 by a team of researchers as a means of standardizing the diagnosis of multiple sclerosis. These criteria, like those for clinically definite multiple sclerosis, require evidence of damage disseminated in space and time. The McDonald criteria also incorporate the use of magnetic resonance imaging to establish the MS diagnosis (Gobbin et al. 2019).

## The structure of myelin

The overall structure of myelin is similar in the PNS and CNS, despite the differences in molecular composition and the fact that CNS myelin is made by oligodendrocytes and PNS myelin by Schwann cells. Myelin is a repetitive multilayer of tightly packed lipid bilayers, which are held together by specific myelin proteins, one of which is MBP. Myelin proteins are among the most long-lived proteins in the body (Toyama et al. 2013; Fornasiero et al. 2018), which reflects the importance of the stability of this macroscopic supramolecular structure to the normal functioning of the nervous system.

**Fig. 1:**
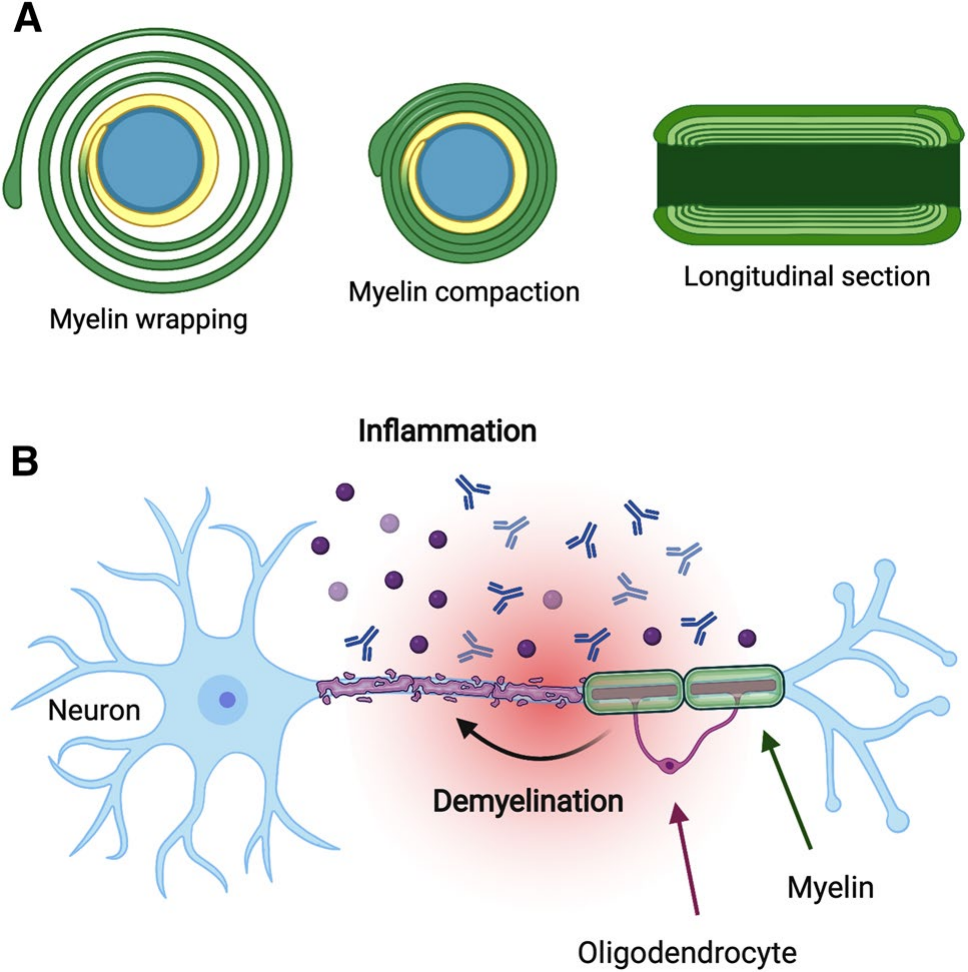
Myelin formation and degradation. **A** During myelination, the myelinating cell wraps its plasma membrane around the axon dozens of times (left). The expression of MBP then induces compaction of this multilayer (middle), into a tightly packed proteolipid devoid of aqueous solvent. The longitudinal section (right) shows a single myelin sheath, bounded by two nodes of Ranvier. **B** During inflammation in an MS plaque, demyelination occurs, leading to neuronal damage and slowing down of nerve impulses, as saltatory conduction is impaired. The figure was prepared using BioRender

During myelination, the myelinating cell wraps its plasma membrane around a selected axon (Fig. 1A) in a dynamic process, which involves growth of the inner tongue of the immature myelin membrane (Snaidero et al. 2014). Upon MBP expression by the oligodendrocyte (Colman et al. 1982), compaction occurs, resulting in the formation of the MBP-rich major dense line as well as the intra-period line at the extracellular apposition. The resulting compact CNS myelin has a very low solvent content, being essentially formed of lipid and protein, while cytoplasmic nano-channels allow for transport of metabolites and signaling (Snaidero et al. 2017).

In MS, inflammation of the CNS targets an immune attack toward the myelin sheath (Fig. 1B). This results in the deterioration of the myelin structure, eventually leading to decreased nerve conduction velocity and neurological symptoms. Re-myelination of the sites of insult can occur via different mechanisms (Neumann et al. 2019; Franklin et al. 2020); this is an intense topic for current research in MS therapy, with a strong focus on stem cell approaches (Genc et al. 2019; Esmaeilizade et al. 2021).

## MBP size isoforms are products of alternative splicing

MBP exists as several size and charge isoforms, some of which have a possible link to the pathogenesis of MS (Vassall et al. 2015). The MBP size isoforms result from alternative splicing of an mRNA transcript (Boggs 2006). The charge isoforms, the focus of the next chapter, are generated by post-translational modifications of a particular MBP size isoform; these modifications decrease the positive charge of MBP and affect its function.

Classic MBP is a product of the Golli (genes of oligodendrocyte lineage) gene complex. Golli gives rise to both the classical MBP and the Golli MBP. Golli MBP proteins are found in the hemopoietic system (Grima et al. 1992), while canonical MBP is thought to only exist in myelin. In humans, the Golli complex has 10 exons, 7 of which give rise to MBP (Givogri et al. 2001). Human MBP consists of 4 different size isoforms of 17.2, 18.5, 20.2, and 21.5 kDa. The 18.5-kDa isoform is the most abundant type in adult human myelin, and by far the most extensively studied. All four isoforms contain domains encoded by exons 1, 3, 4, 6, and 7 (Voskuhl et al. 1993). Domains encoded by exon 2 can only be found in the two largest size isoforms (i.e., 20.2- and 21.5-kDa size isoforms), making these exon 2+ (positive) and the 17.2- and 18.5-kDa size isoforms exon 2− (negative) forms of MBP (Boggs 2006).

The two different subgroups of MBP (i.e., exon 2+ and exon 2−) have been found to dominate in different stages of oligodendrocyte and subsequent myelin development, in both humans and mice (Barbarese et al. 1978). The exon 2+ size isoforms of MBP predominate in early stages of myelination, while exon 2− MBP is characteristic of later stages of myelination and adult mature myelin. The same applies for immature and mature oligodendrocytes, respectively. In humans, the exon 2+ MBP is seen in fetal development and during re-myelination (Gogate et al. 1994). The functional differences between the different size isoforms at the molecular level are currently poorly known.

The discoveries mentioned above gave rise to the hypothesis that exon 2+ MBP might express immunodominant epitopes that are not present in the adult exon 2− MBP. In 1993, Voskuhl et al. published a study on the possible T lymphocyte recognition of an epitope on exon 2+ MBP. At the time, such epitopes were not known. Since there is enhanced expression of exon 2+ MBP transcripts in re-myelination after myelin loss, autoimmunity against an immunodominant epitope on this protein would be of interest in the pathology of MS. Voskuhl et al*.* found that there, indeed, were epitopes on the exon 2+ isoforms recognized by human T cells, thus providing a possible mechanism for progression of MS. In this model, regenerating myelin and immature oligodendrocytes are targets of a T cell-mediated immune response (Voskuhl et al. 1993; Gogate et al. 1994).

## Post-translational modification gives rise to charge isoforms of 18.5 kDa MBP

18.5-kDa MBP exists as 8 different charge isoforms. These are termed C1–C8 and are products of various post-translational modifications, decreasing the net charge of MBP. C1 is the least modified charge isoform of 18.5 kDa MBP, and thus, the charge isoform with the highest positive charge. C2–C6 are modified by, among others, phosphorylation, deamidation, and deimination. C8 is modified by peptidyl arginine deiminase that converts arginine to citrulline (citrullination). This is done for 6–11 arginine residues, with a loss of net positive charge by one for every arginine-to-citrulline conversion (Wood and Moscarello 1989). Like observed for the exon 2+ and exon 2− MBP (see above), the specific charge isoforms seem to dominate at different stages of myelin development and provide different levels of myelin stability. C1, C2, and C3 are considered to be a part of a stable myelin sheath, while C8 is suggested to have a role in development, with a peak in childhood and subsequent decrease in adults. Hence, the C8 charge isoform might be of importance for the formation of myelin rather than its stability (Wood and Moscarello 1989). The focus of this chapter will be on reported changes in proportion of different charge isomers and arginine deimination in MS brain.

The importance of the various charge isoforms of MBP has been studied for decades, and the developmental properties of the C8 isoform have been of interest in research on MS, especially with regard to the association of C8 with destabilized compact myelin. It has been found, when comparing the various charge isoforms of 18.5 kDa MBP in normal human white matter and white matter from an MS patient brain, that there are several differences. A study published in 2003 found that, while the proportion of C2, C3, C4, and C5 did not differ significantly between the two groups, C1 was decreased in MS white matter, while C8 was increased in MS white matter compared to the normal brain (Kim et al. 2003). This could be explained as an indication of attempted re-myelination.

Furthermore, the same study (Kim et al. 2003) found more deiminated arginine residues in MS white matter than in normal white matter, specifically showing that C4 and C5 were deiminated to a greater extent in MS tissue than normal. The study also showed a difference in deimination of C8 between two MS white matter samples. One sample from a mild case of MS showed deimination on several residues, but to a relatively lesser extent. Another sample was from a younger patient with a more aggressive disease and showed extensive deimination of all arginine residues tested. Therefore, a correlation between extent of deimination and severity of disease was postulated (Kim et al. 2003).

At the molecular level, there are many consequences of an increased deimination of arginine residues in MBP. As explained above, MBP loses one net positive charge for each deiminated arginine residue. This results in a less cationic protein, decreased ability to interact with negatively charged lipids, and subsequent decrease in stability and compaction of myelin, further resulting in possible myelin loss (Mastronardi and Moscarello 2005). Second, as reported by Cao et al*.* in 1999, the degradation of citrullinated MBP by cathepsin D (a myelin-associated protease) was much more rapid than that of MBP C1. It was hypothesized that citrulline might have a stabilizing effect on encephalitogenic peptides in MS (Cao et al. 1999). Furthermore, it was observed that the HLA haplotypes associated with greatest genetic risk for MS (HLA-DRB1*15:01 and HLA-DRB5*01:01) preferentially presented peptides that were citrullinated at particular HLA-binding sites (Nguyen and James 2016).

## MBP is an intrinsically disordered protein with propensity to fold locally

Biochemically, MBP has unique properties. It carries a high positive charge, having an isoelectric point of ~ 11. Since the first structural investigations > 50 years ago (Chao and Einstein 1970), a large number of studies have characterized MBP as an IDP (Krigbaum and Hsu 1975; Harauz et al. 2009; Majava et al. 2010; Wang et al. 2011; Raasakka et al. 2017), indicating that MBP is highly flexible and possibly able to interact with multiple binding partners. Indeed, while MBP has been well characterized as a membrane-binding protein (Harauz and Libich 2009; Wang et al. 2011; Vassall et al. 2015; Raasakka et al. 2017), it additionally has a number of protein–protein interaction partners (Baryłko and Dobrowolski 1984; Chan et al. 1990; Roth et al. 1993; Libich and Harauz 2002; Majava et al. 2010; Boggs et al. 2011; Smith et al. 2012). The interplay of these different interactions is important for the correct formation of myelin (Snaidero et al. 2017).

**Fig. 2:**
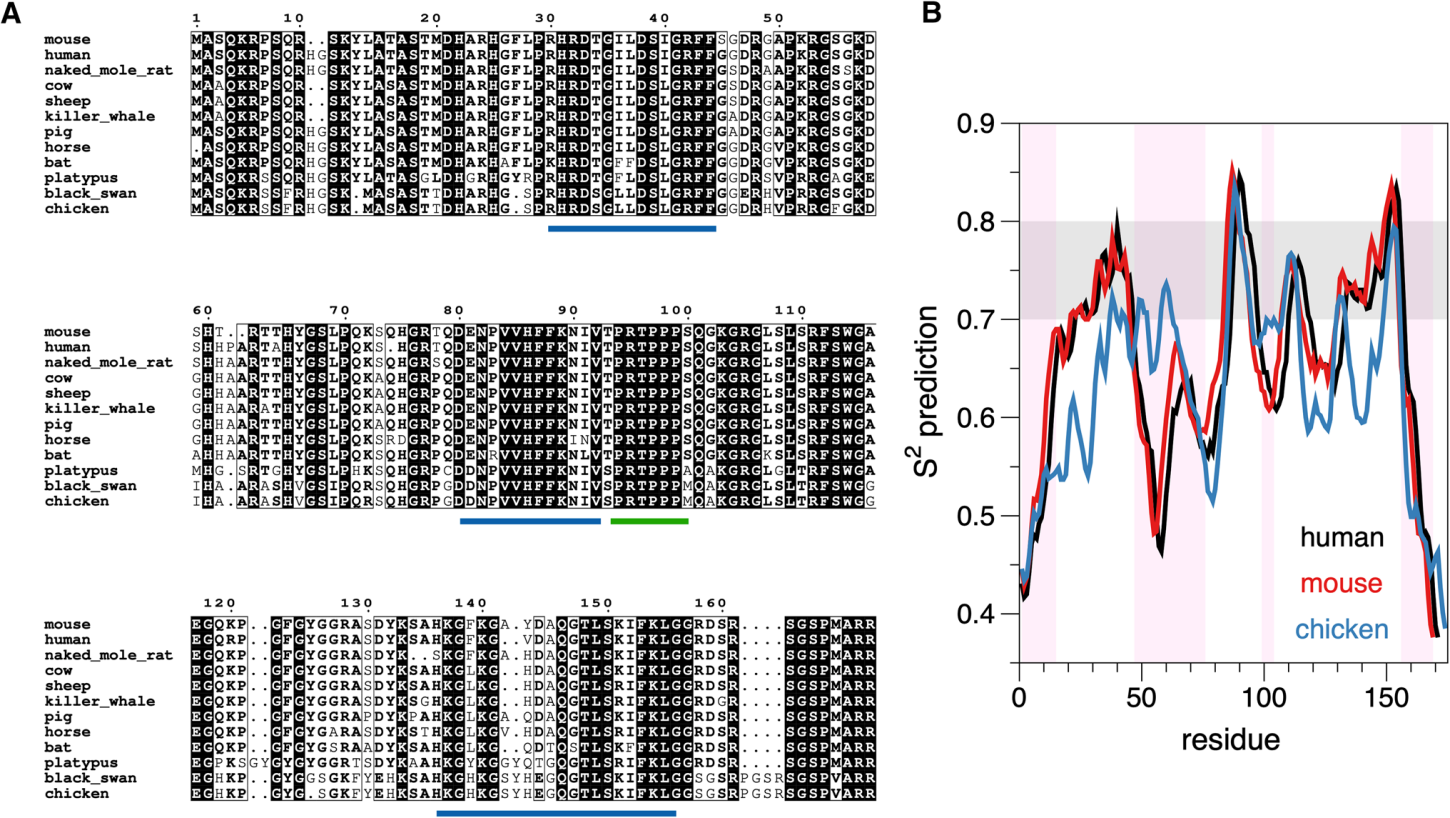
Conservation and intrinsic disorder in MBP. **A** Alignment of MBP sequences from selected tetrapods. Clearly defined conserved segments are detected, which correspond to regions predicted to fold into helices upon molecular interactions with, e.g., lipid membranes or other proteins. The segments marked in blue interact with membranes and/or calmodulin, while the SH3 domain-binding site (Polverini et al. 2008) is indicated in green. The figure was prepared using ESPript (Gouet et al. 1999). B. Bioinformatics analysis of MBP sequences indicates conserved order/disorder between species, and the molecular interaction sites have a propensity to fold into rigid structures. Plotted is the DynaMine (Cilia et al. 2014) flexibility prediction for human, mouse, and chicken 18.5 kDa MBP. Values above 0.8 predict rigid structure, 0.7–0.8 suggests context-dependent folding, and below 0.7, the protein is predicted to be disordered. The shading in pink highlights mouse MBP regions predicted to be disordered by PONDR (Xue et al. 2010)

As the structure of MBP is flexible, without a compact 3D fold, an analysis of MBP sequence conservation can shed light on functionally relevant segments. Selected MBP sequences have been aligned in Fig. 2A, showing strong conservation of certain segments. On the basis of earlier literature, these conserved sites correspond to those binding to the lipid bilayer, or to other proteins. To complement these analyses, bioinformatics-based tools can be used to predict MBP properties (Fig. 2B). The DynaMine prediction (Cilia et al. 2014) suggests 3 regions with a rather rigid structure, possibly being context-dependent. These regions correspond to the known membrane- and/or calmodulin-binding sites. The PONDR prediction of disorder (Obradovic et al. 2003; Xue et al. 2010; Cilia et al. 2014) is in line with the above analysis, showing disordered segments between the membrane-binding sites. The membrane-binding sites are known to fold into helices upon membrane embedment (Harauz et al. 2009; Muruganandam et al. 2013; Raasakka et al. 2017).

Characteristic to myelin compaction in the CNS is the transport of MBP mRNA to oligodendrocytic processes, which are about to compact (Ainger et al. 1993, 1997; Barbarese et al. 1995; Carson et al. 1997). MBP acts as a trigger to glue apposing membranes together (Fig. 1A). Its interaction with lipid surfaces is essentially irreversible, and it forms a brush-like phase onto the membrane surface, which is then attractive toward a second lipid bilayer (Raasakka et al. 2017). Liquid–liquid phase separation appears to play a role in this process (Aggarwal et al. 2013), which eventually produces the major dense line, corresponding to the fusion between two apposing cytoplasmic leaflets of the myelin membrane. MBP is crucial for the formation of the major dense line in the CNS myelin.

Interestingly, the disease mechanisms of amyotrophic lateral sclerosis (ALS) involve mRNA transport and liquid–liquid phase separation, which both are attributes related to MBP in oligodendrocytes. Although ALS is a neuronal disease, oligodendrocytic pathology has been described and linked to a decrease in MBP (Nonneman et al. 2014; Zhou et al. 2017; Wang et al. 2018). The deficiency of oligodendrocytic mRNA transport may cause collateral damage to myelinating cells in addition to neurons per se.

## Animal models for the study of MBP and demyelinating diseases

With regard to MBP, a mouse model, the *shiverer* (Readhead and Hood 1990), is of historical relevance. First described in the 1970s, *shiverer* mice have abnormal CNS myelin, while PNS myelin appears normal (Bird et al. 1978; Dupouey et al. 1979; Privat et al. 1979). At the molecular level, *shiverer* mice were observed to be deficient in MBP (Dupouey et al. 1979). Hence, the *shiverer* mouse is a spontaneous knock-out line for MBP, originating from a time, when current technologies for generating mutant mice were not yet available. The *shiverer *mouse is still a widely used animal model for studying myelination and MBP-related processes therein.

The most widely used experimental animal model for MS is experimental autoimmune encephalomyelitis (EAE). EAE is an immune and neuropathological condition, consisting of CNS inflammation, demyelination, axonal loss, and gliosis, which all are key components of MS pathology (Constantinescu et al. 2011). EAE is induced by stimulating a T-cell-mediated immune response against myelin antigens (Stromnes and Goverman 2006a). There are two forms of EAE, active and adoptive-transfer (AT), caused by different methods of induction. Active EAE is induced by immunizing with an array of tissue and myelin peptides. Among these are MBP and peptides derived from it. AT EAE is induced by immunization of a model animal with myelin-specific CD4^+^ T cells from a donor animal (Stromnes and Goverman 2006b).

EAE is considered a versatile model of neuro-inflammation and demyelination, being used as a model for studying protective mechanisms. A key factor in this versatility is that different induction methods, animal models, and response to pharmacological intervention cause a variety of outcomes. Although this versatility comes with the possibility to tailor the model to a specific study, such as modeling different forms of MS, it also causes problems for the translation from an animal model of MS to the actual disease in humans (Constantinescu et al. 2011).

Several of the disease-modifying therapies (DMT) for MS have been, at least partially, related to studies on EAE. A DMT termed GA (glatiramer acetate), trade name Copaxone, is one of these, with a tight link to EAE. GA is a copolymer consisting of a specific ratio of the amino acids tyrosine, glutamine, alanine, and lysine, and it physico-chemically resembles MBP (Stapulionis et al. 2008; Jalilian et al. 2012). GA was first developed by Teitelbaum et al*.* in 1971 as a potential encephalitogen, but it instead turned out to be an effective blocker of EAE (Teitelbaum et al. 1971).

Altered peptide ligands (APL) of MBP have been studied as treatments for EAE, the concept being that substitution of one or more amino acids of the APL would interfere with MHC or T cell receptor-binding properties and cause tolerance to the native peptide through various mechanisms (Constantinescu et al. 2011). An in vivo study on this hypothesis showed tolerance in a murine model immunized with a particular MBP-specific T cell clone (Brocke et al. 1996). This same theory was tested in MS patients, but the trial was discontinued, because 9% of patients developed hypersensitivity reactions. Another phase II clinical trial found that MBP APL could lead to exacerbation of MS. This led to two conclusions: one being the confirmation of a link between autoimmunity toward MBP and MS. The second conclusion was that EAE as a model for MS is highly relevant, but great care should be taken to understand underlying mechanisms, when extrapolating from EAE to MS and considering specific immunotherapies (Bielekova et al. 2000; Constantinescu et al. 2011).

## Anti-myelin antibodies as predictors for the development of multiple sclerosis after a first demyelinating event

Anti-myelin antibodies (anti-MOG and anti-MBP) have long been targets of interest in MS research, and some foci of interest have been the relevance of anti-MBP antibodies as a possible cause of MS, as well as anti-myelin antibodies as markers for severity of disease or as markers for risk of progression. Many of the answers to these research questions remain uncertain, and conflicting results exist. Furthermore, serum IgG antibodies against MBP were shown to potentially distinguish ADEM from MS (Van Haren et al. 2013). In this chapter, we will look closer at the possible clinical use of testing for serum anti-myelin antibody status in patients, who experience a first demyelinating event associated with MS.

After a first demyelinating event, there is an elevated risk of developing MS, but the outcome for the individual is uncertain. This demyelinating event is called a clinically isolated syndrome (CIS) (Miller et al. 2012), and most MS patients present with such an event (Villar et al. 2011). CIS is defined as a single demyelinating event affecting the central nervous system; the episode must last at least 24 h, and there must not be any association to other organic disease (fever, infections, metabolic disorders, etc.). The increased long-term risk of the patient for developing MS depends on the detection of additional brain lesions on magnetic resonance imaging (MRI) scans. When CIS is accompanied by such findings, and the lesions are similar to those found in MS, the patient has a 60–80% risk of a second demyelinating event and an MS diagnosis. For patients with a normal MRI scan, except for the symptomatic lesion, the risk for progression is ~ 20% (Miller et al. 2012).

Kuhle et al*.* published in 2007 an article on establishing anti-MOG and anti-MBP antibody status in patients with CIS and its predictive value for progression to clinically definite MS or a diagnosis of MS as defined by the McDonald criteria (Kuhle et al. 2007b). In this study, anti-myelin antibody status was established for 462 patients, whereby 52% and 36% tested positive for anti-myelin IgM and IgG antibodies, respectively. The rest of the patients tested negative for both anti-MOG and anti-MBP, both IgM and IgG. The study was done on patients recruited to the BENEFIT trial (Betaferon in Newly Emerging Multiple sclerosis for Initial Treatment), a trial that studied the effect of interferon beta-1b on patients with CIS.

Earlier results suggested an association between serum anti-myelin antibody status and prognosis for patients after a first demyelinating event, and that analysis of anti-MOG and anti-MBP antibody status could be used to estimate individual risk for progression of disease (Berger et al. 2003). Kuhle et al*.* however, found no “increase in the risk of clinically definite multiple sclerosis or of multiple sclerosis according to the McDonald criteria among patients who were positive for anti-MOG antibodies, anti-MBP antibodies, or both” (Kuhle et al. 2007b). This was the conclusion for not just the total study population, but for all subgroups analyzed. Accordingly, while anti-MOG and anti-MBP antibodies are correlated to inflammatory signs in MS patients, their prognostic value for predicting MS progression is questionable (Kuhle et al. 2007a).

The above findings are supported by a cohort study conducted by Pelayo et al*.* (Pelayo et al. 2007). In addition to investigating the association between serum anti-myelin antibody status and rate of progression to clinically definite multiple sclerosis, the group looked at the association regarding time to conversion for patients, who converted to clinically definite MS during the follow-up period (mean of 46.7 ± 21.2 months). The conclusion, like that of Kuhle et al. (2007b), was that there were no significant differences between anti-myelin antibody-positive patients and anti-myelin antibody-negative patients in the rate of conversion. Likewise, for patients progressing to clinically definite MS during the follow-up period, no significant difference was found in the median time to conversion between the two groups (Pelayo et al. 2007).

It would, thus, seem that the clinical relevance of establishing a patient’s serum anti-myelin antibody status for the purpose of evaluating the individual risk for progression to clinically definite MS after a CIS is, at this time, limited and uncertain. Many other possible markers for risk of disease progression have been investigated with varying results. Among these are transcription factors, cell membrane receptors, and cytokines, particularly those involving immune cells.

One marker showing promising results in a study published in 2011 was CD5^+^ B cells (Villar et al. 2011). It is known that these cells are involved in certain types of autoimmune disease. The study found that an increase in the percentage of CD5^+^ B cells in blood was associated with a higher risk of progression from CIS to clinically definite MS. It was postulated that this marker, therefore, could be useful for improving the efficacy of the prognosis for individual patients, but that further investigation on a larger scale is required (Villar et al. 2011).

A biomarker for the risk of progression in patients with CIS would have great clinical value, both to initiate drug therapy and to combat the uncertainty of outcome for the individual patient. A marker found in serum would be preferred over a marker in the CSF, considering the risk of harvesting test samples. While putative biomarkers have been found in small-scale studies, more investigation is needed on this topic.

## Cross-reactivity caused by similarities between viral antigens and MBP epitopes

Cross-reactivity to exogenous antigens and self-antigens, termed molecular mimicry, has been a proposed mechanism, causal and exacerbating, of many autoimmune diseases. This builds on the theory that exposure to a foreign antigen with a similar amino acid sequence or structure to host antigens can cause an autoimmune response. Examples of diseases, in which this is seen as a plausible cause, are heart damage after rheumatic fever with *Streptococcus pyogenes* infection and the association between *Campylobacter jejuni* and Guillan-Barré syndrome (Cusick et al. 2012), an autoimmune demyelinating condition of the PNS. This chapter will discuss molecular mimicry related to MBP as a putative mechanism in the pathogenesis of MS. It should be noted that many other theories on the pathogenesis of MS exist, being a complex chronic inflammatory disease, including autoimmune reaction against GDP-l-fucose synthase (Planas et al. 2018), as well as both environmental and genetic factors (Ghasemi et al. 2017). The various factors involved have recently been reviewed in detail elsewhere (Gasperoni et al. 2019).

The hypothesis of molecular mimicry related to microbial and viral pathogens in MS pathogenesis has long been popular, especially related to the Epstein-Barr virus (EBV), and some of the research conducted on the subject has focused on MBP itself as a possible target autoantigen. It has been proven that cross-reactions between EBV and MBP do exist, but there are also cross-reactions between MBP and other viruses. There is a strong correlation between MS and EBV, with EBV infection in adolescence and young adulthood being one of the best-known environmental risk factors in MS (Guan et al. 2019). Therefore, as stated by Füst in 2011, “a cross-reaction itself can hardly be responsible for the relationship between EBV and MS” (Füst 2011).

In a study that structurally aligned single-chain variable fragments (scFv) from blood lymphocytes of MS patients selected toward MBP (Gabibov et al. 2011), high homology was found between the variable regions of CSF MS-associated antibodies and antibodies toward EBV latent membrane protein 1 (LMP1). One scFv clone showed natural cross-reactivity, reacting with both MBP and LMP1 in vitro. The authors concluded that antibodies induced against LMP1 during EBV infection might act as an inflammatory trigger by reacting with MBP, thereby suggesting molecular mimicry as a direct mechanism in the pathogenesis of MS (Gabibov et al. 2011).

A more recent study looked at the humoral immune response toward EBV nuclear antigen-1 (EBNA-1) in MS patients (Jog et al. 2020). A qualitative difference was found between the anti-EBNA-1 antibody in MS patients and healthy controls, stemming from the recognition of unique epitopes in MS patients that were not recognized in the control group. The authors saw that anti-EBNA-1 found in MS patients had, unlike that from the controls, cross-reacted with MBP. The antibody that cross-reacted was anti-EBNA_411-426_. When a murine model was immunized with EBNA_411-426_ peptides, varying levels of the clinical symptoms of EAE were observed. This led to the conclusion that the cross-reactive sequence of EBNA-1 can cause an anti-MBP response in mice (Jog et al. 2020).

## MBP itself as a biomarker of myelin damage

In addition to anti-MBP antibodies, the MBP protein per se has been studied and used as a possible biomarker for brain tissue injury and neurodegenerative disease, both in serum and in the CSF (Barkhof et al. 1992; Katsavos and Anagnostouli 2013; Zavialova et al. 2017; Kim et al. 2018). Being an integral component of compact myelin, the detection of MBP in the CSF is a direct sign of myelin breakdown. In line with this, MBP and its fragments can be detected in the CSF of most MS patients during relapse (Lamers et al. 1998; Sellebjerg et al. 1998). However, it has been suggested that the added value of testing the CSF for MBP in MS diagnosis is low (Greene et al. 2012). In addition to MS, MBP has been detected in the CSF in a number of neurodegenerative conditions, including ADEM (Re and Giachetti 1999; Koshihara et al. 2014), encephalitis (Jacque et al. 1982), acute cerebral infarction (Strand et al. 1984), and neuro-Behcet's disease (Ohta et al. 1980). The presence of MBP in serum or the CSF is an indication of myelin damage in general, and testing for it may be applicable to diagnosis of different neurodegenerative disease states, including spinal cord demyelination (Ohta et al. 2002).

## Concluding remarks

Multiple sclerosis is a devastating chronic disease with an unknown mechanism of pathogenesis at the molecular level. One of the leading theories currently is that exposure to foreign antigens, in combination with environmental and genetic factors, causes a cross-reactive T cell-mediated immune response in susceptible individuals. A possible autoantigen target for this cross-reactive immune response is MBP and peptides derived from it, leading to demyelination and onset of disease. Getting a clearer understanding of the causes of MS, as well as identifying reliable biomarkers, would have a large impact on the treatment and prevention of MS and, thus, of great relevance for individuals at high risk or already affected by MS. However, more research will, indeed, be needed to reach such a goal.

## Data Availability

All data are freely available upon request.

## Declarations

### Research involving human and animals participants

No research involving humans or animals was carried out.
